# A Massive Open Online Course to Educate Healthcare Professionals & Caregivers About Alzheimer’s Disease

**DOI:** 10.1093/geroni/igab046.2797

**Published:** 2021-12-17

**Authors:** Mary DiBartolo

**Affiliations:** Salisbury University, Salisbury, Maryland, United States

## Abstract

Over 6 million Americans and 50 million persons worldwide are estimated to have Alzheimer’s disease (AD) as it remains the major cause of dementia in the older adult population. Both healthcare professionals and family caregivers struggle with the complexities of caring for individuals with this progressive neurological disease. To address the ongoing knowledge and care gap regarding Alzheimer’s disease among both healthcare professionals and family caregivers, a comprehensive massive open online course (MOOC) was developed and made available via the edX platform. MOOCs are open access and interactive courses offered via the web; they have emerged as a popular, self-paced mode of distance learning. Launched in 2020, the MOOC titled, Alzheimer’s Disease & Dementia Care, consists of five modules reviewing symptoms, diagnosis, medications, communication and care tips, as well as a module outlining special considerations when the person with AD is hospitalized. While this educational strategy targets healthcare professionals (such as nurses, physical therapists and related practitioners), it is also designed for lay caregivers or anyone who wants to learn more the disease. The course utilizes a variety of teaching modalities and is free. To date over 7000 persons have enrolled from over 140 countries. MOOCs remain an innovative and engaging educational strategy to reach a global audience. More importantly, they can serve as an another outlet to enhance both the competence and confidence of both healthcare professionals and family caregivers by sharing best practices in caring for those with Alzheimer’s disease and related dementias.

